# The Importance of Serial POCUS Exams – Dual Pathologies in Play 

**DOI:** 10.24908/pocus.v8i2.16595

**Published:** 2023-11-27

**Authors:** Rahul Nair, Jonathan Zuo, Ariel L Shiloh

**Affiliations:** 1 Division of Critical Care Medicine, Department of Medicine, Montefiore Medical Center/Albert Einstein College of Medicine Bronx, NY USA; 2 Albert Einstein College of Medicine Bronx, NY USA

**Keywords:** Tamponade, Takotsubo, Pericardial effusion, Butterfly iQ, Cardiac POCUS, POCUS, assessment Case

## Abstract

Serial point of care ultrasound (POCUS) exams are essential to assess acute pericardial effusions which can rapidly evolve into cardiac tamponade. A typical presentation includes dyspnea, tachycardia, and chest pain. Importantly, serial cardiac exams in such high-risk patients can detect other concurrent pathologies. We present an unusual case of a patient who initially presented with an acute circumferential pericardial effusion and upon serial POCUS exams developed an unexpected Takotsubo cardiomyopathy in the setting of cardiac tamponade.

## Case

A 63-year-old woman with a past medical history of Human Immunodeficiency Virus, asthma, and previously treated Hodgkin’s lymphoma presented to the emergency department with 3 weeks of worsening shortness of breath. On initial assessment in the emergency department, the patient was hemodynamically unstable with a heart rate of 140 beats per minute (bpm) and a blood pressure of 88/68 mm Hg. Bedside point of care ultrasound (POCUS) was performed and demonstrated a moderate circumferential pericardial effusion with normal left ventricular function and a collapsible inferior vena-cava (IVC). Intravenous fluids were initiated with resolution of the patient’s hemodynamic instability (Video S1). A computer tomography (CT) of the chest was also obtained in the emergency department which showed extensive mediastinal lymphadenopathy with right middle lobe consolidation. The patient was admitted for evaluation of the pericardial effusion and CT findings. 

Forty-eight hours after admission, the patient reported persistent midsternal chest pain and shortness of breath with a blood pressure of 110/60 mm Hg, heart rate of 127 bpm, and an oxygen saturation of 100% on 2-liters nasal cannula. Repeat troponin I was elevated at 1.02ng/ml (ref - <0.03 ng/ml). POCUS was performed which revealed mid-to apical left ventricular (LV) akinesis and an interval reduction of LV function along with right atrial systolic collapse, right ventricular diastolic collapse and a plethoric IVC (Video S2). Limited transthoracic echocardiogram done by the echocardiography lab shortly after confirmed the findings. These findings were concerning for Takotsubo/stress induced cardiomyopathy in the setting of tamponade physiology. 

The patient underwent a pericardiocentesis with removal of 240 mL of fluid with improvement of tachycardia and dyspnea. Formal echocardiography 48 hours after the procedure showed minimal residual effusion with improvement of LV function, which returned to normal within a week (Video S3). Cytology of the pericardial fluid showed inflammatory and mesothelial cells with no evidence of malignant cells. However, subsequent lymph node biopsy revealed recurrence of Hodgkin lymphoma to which her pericardial effusion was attributed. 

## Discussion

Cardiac tamponade is a pericardial syndrome characterized by impairment of diastolic filling of the ventricles causing reduction of cardiac output. The classically advanced signs of tamponade described as hypotension, distension of jugular veins, and diminished heart sounds (Becks Triad) are present in a minority of patients. The most common initial presenting symptoms are dyspnea and tachycardia, which may be present in the absence of hypotension 1. 

Echocardiographic evaluation of tamponade includes: 

i) Quantity and quality of pericardial fluid 

ii) Systolic right atrial collapse 

iii) Diastolic right ventricular size and variability with the respiratory cycle 

iv) Interventricular septal shift of the left ventricle during inspiration 

v) Collapsibility of IVC 2, 3. 

Diastolic right ventricular collapse is specific for tamponade while IVC plethora is highly sensitive. Systolic right atrial collapse is often the earliest sign of tamponade. Comprehensive or advanced critical care echocardiography can be used to detect exaggerated respiratory cycle changes in mitral and tricuspid valve in-flow velocities as a surrogate for pulsus paradoxus 2. Ideally electrocardiographic gating is used to delineate systole and diastole but this not often routine with POCUS. 

In this patient the initial POCUS exam revealed a moderate sized pericardial effusion with a fully collapsible IVC making tamponade physiology less likely. When the patient developed new onset chest pain and shortness of breath this raised the concern for worsening tamponade. A repeat ultrasound revealed an unexpected finding of stress induced cardiomyopathy which can potentially be attributable to progressive tamponade development. Potentially the tamponade was further exacerbated by left ventricular dysfunction.In dog models right atrial and ventricular collapse occurred with significantly smaller volumes of pericardial fluid in the setting of induced left ventricular dysfunction 4. This could certainly lead to a vicious cycle of LV systolic dysfunction and worsening tamponade physiology leading to acute decompensation. 

Takotsubo-associated myocardial dysfunction is irrespective of vascular territories, and commonly presents as transient mid to apical akinesia, hypokinesia, or dyskinesia in the absence of obstructive coronary disease. Other findings include circumferential apical dilatation (apical ballooning), basal hyperkinesia and a severely reduced left ventricular function 5. Treatment of Takotsubo cardiomyopathy is mainly supportive, however complications that arise such as Left Ventricular outflow tract obstruction (LVOT) (20%), cardiogenic shock (12.4%), thrombus formation (8%) all will have differing managements^6^, ^7^. Drainage of the pericardial effusion improved the patient’s hemodynamic status and led to the resolution of the cardiomyopathy, favouring a diagnosis of Takotsubo cardiomyopathy. Ideally, a true diagnosis of Takotsubo requires cardiac catheterization as the patient’s troponin plateaued at 1ng/ml cardiac catheterization was not pursued at the time by the inpatient cardiology service 8. 

## Conclusion

This case highlights the importance of serial cardiac POCUS examinations in the evaluation of pericardial effusion. Additionally, it highlights the importance of recognizing an acute concurrent cardiac pathology that can quickly lead to a vicious cycle of cardiac dysfunction leading to decompensation. 

## Statement of Consent

The authors confirm that consent to publish this case was obtained from the patient.

## Disclosures

All authors of this manuscript have no conflicts of interest. The authors listed have no affiliations with or involvement in any organization or entity with any financial interest in the subject matter or materials discussed in this manuscript.

## Supplementary Material

 Video S1 Cardiac POCUS demonstrating pericardial effusion

Video S2 Cardiac POCUS demonstrating takotsubo cardiomyopathy

Video S3 Post pericardiocentesis demonstrating improved left ventricular function
